# A three-arm randomised controlled trial to evaluate the efficacy of a positive psychology and social networking intervention in promoting mental health among HIV-infected men who have sex with men in China

**DOI:** 10.1017/S2045796021000081

**Published:** 2021-03-19

**Authors:** J. Li, P. K. H. Mo, C. W. Kahler, J. T. F. Lau

**Affiliations:** 1 School of Public Health, Sun Yat-Sen University, Guangzhou, China; 2 Division of Behavioral Health and Health Promotion, The School of Public Health and Primary Care, Faculty of Medicine, The Chinese University of Hong Kong, Shatin, Hong Kong; 3 The Chinese University of Hong Kong Shenzhen Research Institute, Shenzhen, China; 4 Department of Behavioral and Social Sciences, Brown University School of Public Health, USA; 5 Centre for Medical Anthropology and Behavioral Health, Sun Yat-sen University, Guangzhou, China

**Keywords:** Positive psychology, men who have sex with men, randomised controlled trial, depression, China

## Abstract

**Aims:**

There is a lack of mental health promotion and treatment services targeting HIV-positive men who have sex with men (HIVMSM) in China. The aim of this study was to evaluate the mental health promotion efficacy of an online intervention that combined Three Good Things (TGT) with electronic social networking (TGT-SN) and an intervention that used TGT only (TGT-only), compared with a control group.

**Methods:**

We conducted a randomised controlled trial among HIVMSM in Chengdu, China. The participants were randomly assigned to the TGT-SN, TGT-only, and control groups. The participants in the TGT-SN group were divided into five social network groups and asked to post brief messages to the group about three good things that they had experienced and for which they felt grateful. The participants in the TGT-only group were only required to write down their three good things daily without sharing them with others. The control group received information about mental health promotion once a week for a month. The primary outcome was probable depression. Secondary outcomes were anxiety, positive and negative affect, gratitude, happiness and social support. These outcomes were assessed at baseline, 1, 3, 6 and 12 months after the intervention. Repeated-measures analyses were conducted using generalised estimation equations. The study was registered with the Chinese Clinical Trial Registry (ChiCTR-TRC-13003252).

**Results:**

Between June 2013 and May 2015, 404 participants were enrolled and randomly assigned to either the TGT-SN (*n* = 129), TGT-only (*n* = 139) or control group (*n* = 136). The main effects of TGT-SN (adjusted odds ratio (aOR) = 0.75, 95% CI 0.52–1.09; *p* = 0.131) and TGT-only (aOR = 0.83, 95% CI 0.57–1.21; *p* = 0.332) in reducing depression were statistically non-significant. The participants of the TGT-SN group showed significantly lower anxiety symptoms (aOR = 0.62, 95% CI 0.43–0.89; *p* = 0.009) and negative affect (*β* = −1.62, 95% CI 2.98 to −0.26; *p* = 0.019) over time compared with those of the control group. No significant main effect was found for any secondary outcomes for the TGT-only group.

**Conclusions:**

The novel intervention combining the TGT exercise with electronic social networking was found effective in reducing anxiety and negative affect among HIVMSM.

## Introduction

In China, the HIV epidemic among men who have sex with men (MSM) is worsening in all regions (Zhang *et al*., 2013). In addition to life-threatening physical illnesses, HIV-positive men who have sex with men (HIVMSM) are exposed to severe stigma and numerous types of stressors, such as those related to relationship conflicts, finance and family problems (Thompson *et al*., 1996; Berg *et al*., 2008), resulting in a high prevalence of mental health problems. The prevalence of depression among HIVMSM ranges from 35% to 49%, which is higher than that of people living with HIV (PLWH) in general and that of HIV-negative MSM (Mills *et al*., 2004; Comulada *et al*., 2010; Bogart *et al*., 2011; Sivasubramanian *et al*., 2011). Previous studies also have reported a high prevalence of anxiety symptoms among HIVMSM in China, ranging from 13.0 to 32.1% (He *et al*., 2012; Wu *et al*., 2014).

Psychological problems among PLWH have been shown to be significantly associated with a number of negative HIV-related health outcomes and high-risk behaviours, such as low service utilisation rate, poor adherence to antiretroviral therapy (ART), faster progression to AIDS and shorter survival (Perry and Fishman, 1993; Lyketsos *et al*., 1996; Cook *et al*., 2002; Schuster *et al*., 2012), substance use, poor self-care (Gordillo *et al*., 1999; Catz *et al*., 2000; Langebeek *et al*., 2014) and risky sex (Parsons *et al*., 2003), some of which potentially contribute to HIV transmission. There is, however, a large gap in mental health services, as most of the health workers serving PLWH in China are clinicians who have not been trained in psychology and counselling (Xiang and Wu, 2010). There is a large shortage of psychiatrists and clinical psychologists in China in general (Jacob *et al*., 2007; Gao *et al*., 2010), and mental health support and treatments for PLWH, including HIVMSM, are limited in most parts of the country (Zhao *et al*., 2009; Tao *et al*., 2010). Effective, sustainable and low-cost mental health promotion interventions for HIVMSM are greatly needed.

Positive psychology-based approaches to mental health promotion have drawn increasing attention and empirical support. Positive psychology emphasises factors that are protective of mental health problems, such as optimism (Ironson *et al*., 2005), positive affect (Moskowitz, 2003; Lyubomirsky *et al*., 2005a) and positive behaviours (e.g. expressing emotions and proactive coping). A variety of evidence-based positive psychological interventions (PPI), such as Random Acts of Kindness, Gratitude Visit, Using Signature Strengths and Three Good Things (TGT), have been developed (Emmons and McCullough, 2003; Lyubomirsky *et al*., 2005b; Seligman *et al*., 2005; Sheldon and Lyubomirsky, 2006). Such interventions have been shown to be effective in reducing mental health problems and enhancing well-being among many diseased and non-diseased populations. This study used a TGT intervention, in which the participants were asked to write down three things that went well every day to express gratitude towards life in general. TGT is relatively easy to implement and has been widely used (Emmons and McCullough, 2003; Lyubomirsky *et al*., 2005b; Seligman *et al*., 2005).

Previous studies have suggested that social networks can act as a social/peer support system to promote mental health at a low cost (Griffiths *et al*., 2006; Proudfoot *et al*., 2012). It has further been shown that happiness can be ‘transmitted’ from one person to another, up to three degrees of association, within social networks (Fowler and Christakis, 2008). A specific aim of this study was to test whether the effects of PPI could be enhanced by combining PPI with social networking. There are several social networking systems in mainland China. Tencent QQ (known as QQ) is one of the most widely used and has multiple functions, such as one-to-one and group chat, blogs, and sharing photos, videos, notes and links. In Chengdu, China, it is estimated that over 85% of HIVMSM are using QQ. In this study, the intervention was thus implemented via QQ.

This study carried out a three-arm randomised controlled trial to evaluate the efficacy of an online intervention that combined TGT with electronic social networking (TGT-SN) and an intervention that used TGT only (TGT-only), compared with a control group that received weekly emails containing information on promoting mental health. We hypothesised that participation in TGT-SN and TGT-only, compared with a control group receiving information only, would result in lower depressive symptoms, anxiety symptoms, and negative affect, and higher levels of happiness, gratitude and positive affect, during the 12-month follow-up period. It was also hypothesised that the effect of TGT-SN on these outcomes would be stronger than that of TGT-only.

## Methods

### Participants

The participants were recruited in Chengdu, China, by four well-trained peer fieldworkers, who were staff of a local non-governmental organisation (NGO). The NGO was one of the largest gay organisations in China. The inclusion criteria were the following: (1) men who have had anal sex with at least one man in the last 6 months, (2) over 18 of age, (3) diagnosed as HIV-positive at least 3 months prior (as newly diagnosed PLWH tends to be unstable), (4) intending to stay in Chengdu for the coming 6 months, and (5) being a regular QQ user (i.e. using QQ at least once a week).

The exclusion criteria were as follows: (1) the presence of severe AIDS symptoms or other medical conditions, (2) having severe depression or suicidal ideation, (3) sharing their QQ account with others, and (4) utilising psychiatric services or psychological counselling or participating in other interventions at the time of recruitment or during the study period. The Patient Health Questionnaire (PHQ) (Bian *et al*., 2011) was used to screen for severe depression and suicidal ideation. The PHQ contains nine items, including one on suicidal ideation. Each item was rated on a four-point Likert scale, ranging from 0 = not at all to 3 = almost every day. A total score of 20 or higher represents the threshold for severe depression. In this study, those who scored 20 or higher, or those who scored 3 on the item on suicidal ideation, were excluded and referred to health care professionals.

### Procedure

The participants were recruited from June 2013 through to May 2015, and follow-ups were conducted from September 2013 through to July 2016. The study was publicised through posters displayed in the public area of the NGO and on the social media of its service users. Four peer field workers approached prospective participants by making phone calls and/or reaching them through social media, briefed them about the study and logistics, and invited them to participate in the study after confirming their eligibility. Interested participants were invited to meet with the peer fieldworkers at the office of the NGO. The participants were informed that refusal would not affect their right to use any services and they could quit at any stage of the study without needing to give a reason. With written informed consent, anonymous face-to-face baseline interviews were conducted in a private room, using a pilot-tested and structured questionnaire that took about 20 min to complete. Upon completion of the baseline interview, monetary compensation of RMB 50 (about US$6) was given to each participant for their time. The trial was prospectively registered on the Chinese Clinical Trial Registry (ref.: ChiCTR-TRC-13003252). Ethical approval was obtained from the joint Chinese University of Hong Kong-New Territories East Cluster Clinical Research Ethics Committee (reference number: CRE-2013.503) and the Survey and Behavioural Research Ethics Committee of the Chinese University of Hong Kong.

**Fig. 1.**
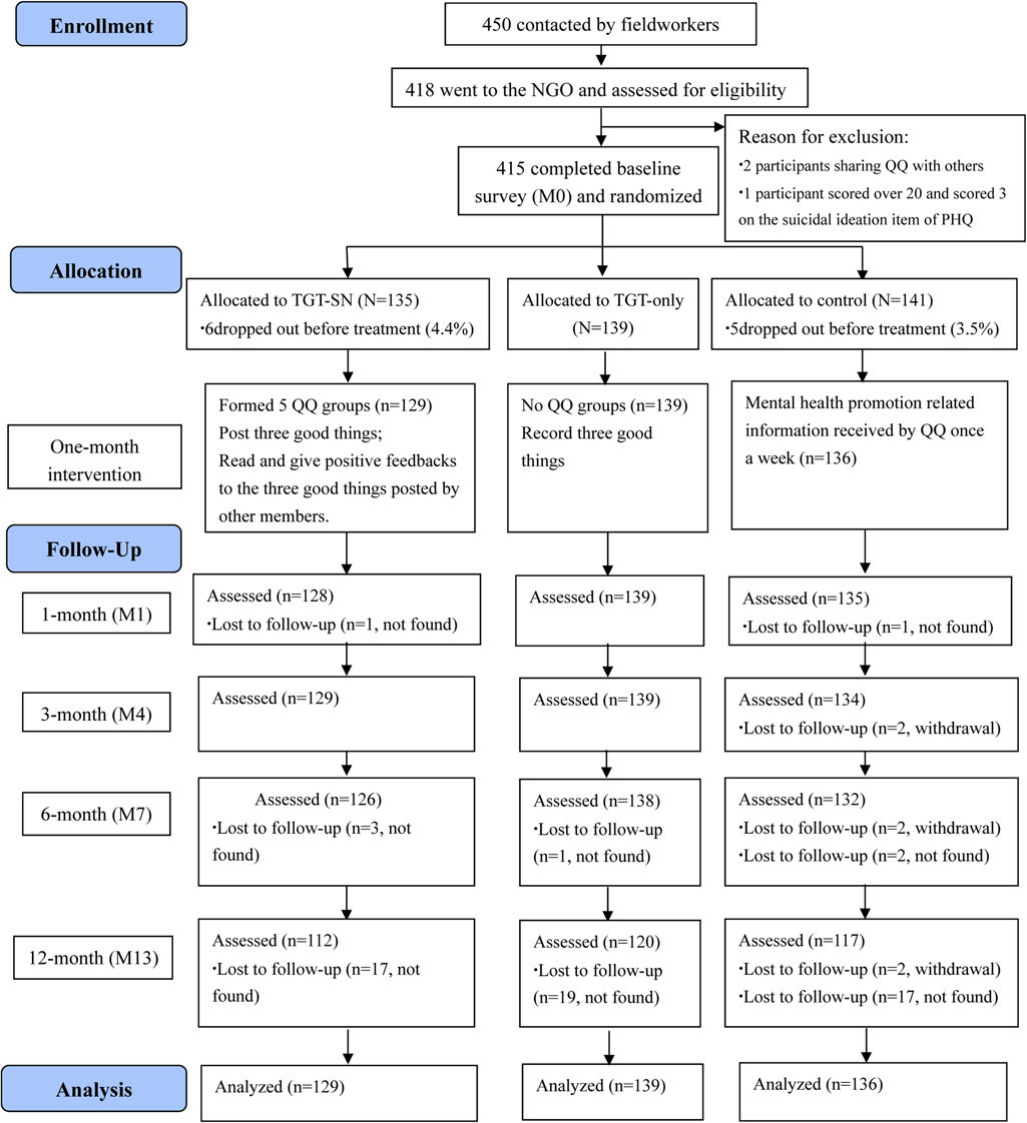
The CONSORT flow diagram of the study.

There were about 1000 HIVMSM identified in Chengdu (Chengdu Tongle Health Counseling Service Center, 2007; Feng *et al*., 2010). The local NGO served about 600 of them at the time of the study and could potentially contact them by phone calls and through social media. A total of 450 of them were successfully contacted by peer fieldworkers and invited to participate in this study; among them, 418 were interested and attended an eligibility assessment. Three participants did not meet the inclusion/exclusion criteria (two participants were sharing their QQ accounts with others and one participant scored over the severe depression threshold of 20 and scored 3 on the suicidal ideation item of the PHQ at baseline). Therefore, 415 (92.2%) participants provided written informed consent and completed the baseline assessment (M0); these were randomised into three groups. The Consolidated Standards of Reporting Trials (CONSORT) flow diagram of this study is shown in Fig. 1.

#### Randomisation

Block randomisation (size = 6) was used, and randomisation was performed using a computerised random number generator. Allocation was concealed from all participants and field workers by using sealed and sequentially numbered envelopes. After randomisation, all participants were provided with a manual to explain the details of the intervention that they were assigned to. The TGT-SN, TGT-only and control groups had 135, 139 and 141 participants assigned to them, respectively. After randomisation but prior to the commencement of the interventions, 11 participants dropped out of the study as they did not add the study's QQ account; they were not followed-up or included in outcome analyses. The analytic sample therefore comprised 404 participants (*n* = 129 for TGT-SN; *n* = 139 for TGT-only; *n* = 136 for the control group).

#### Assessment

All participants were asked to visit the NGO to complete face-to-face follow-up assessments at the completion of the 1-month intervention (M1), 3-month post-intervention (M4), 6-month post-intervention (M7) and 12-month post-intervention (M13). Upon completion of each of the follow-up interviews, monetary compensation of RMB 50 (about US$6) was provided to the participants for their time.

### Treatment conditions

#### TGT-SN group

The participants in the TGT-SN group were divided into five ‘QQ groups’, each with 11–30 persons. During the 1-month intervention period, the participants in TGT-SN were asked to: (1) post brief messages to the group every day three good things that they had experienced and for which they felt grateful, (2) read the three good things posted by other members, and (3) provide at least three feedbacks on the posted messages of others each day through comments or ‘Likes’. The research assistants sent feedback in the form of two ‘Likes’ to each member every week on different weekdays, to ensure everyone was receiving some positive feedback. A template of the intervention process and examples of the three good things were provided to members of this intervention group.

Some ground rules were set for the TGT-SN group and explained to the participants before obtaining their consent to join the study. The participants were requested: (1) not to release any personally identifying information (e.g. name, photo, personal email address and workplace) to other participants to maintain anonymity and protect their privacy, (2) not to copy the messages to other people (to avoid contamination of the control group), (3) to post only supportive messages and not to air negative views or personal problems in the messages, (4) not to have social conversations beyond the messages related to the three good things and the associated feedback, and (5) not to release the study's QQ account number to others. Research assistants monitored the process, removed inappropriate messages and solved problems that arose during the intervention process.

#### TGT-only group

The participants in the TGT-only group were asked to write down each day three good things that they experienced and for which they felt grateful. They did not need to share their three good things with others, so no QQ group was formed.

#### Control group

No QQ group was formed for the control group. The members of this group received information about mental health promotion from the research assistants via personal QQ messages sent once a week during the 1-month intervention period. The materials were prepared by a health psychologist in consultation with the director of the NGO. There was no interaction between the research assistants and the participants in the control group, nor was there any interaction among the members.

### Measurements

Socio-demographic information was collected, including age, gender, education level, employment status, income, marital status and sexual orientation. The participants were also asked about HIV-related background characteristics, including stage of HIV/AIDS (i.e. asymptomatic HIV infection and AIDS), CD4 testing behaviour (i.e. ‘Have you ever taken a CD4 test?’), disclosure of HIV status to their sex partners (disclosure status to male regular partners, male non-regular partners and female sexual partners) and self-perceived health status (from 1 = *very poor* to 5 = *very good*).

#### Primary outcome

*Depression.* The 20-item Center for Epidemiologic Studies Depression Scale (CES-D) (Cheung and Bagley, 1998) was used to assess the presence of depressive symptoms. The Chinese version of the CES-D has been validated with good internal reliability (Chi and Boey, 1993; Cheung and Bagley, 1998; Song *et al*., 2008). Cronbach's *α* for CES-D ranges from 0.84 to 0.91 among PLWH (Richardson *et al*., 2001; Hudson *et al*., 2004; Farley *et al*., 2010; Chishinga *et al*., 2011) and from 0.85 to 0.92 among MSM (Alvy *et al*., 2011; Berg *et al*., 2011; Tucker *et al*., 2014). The participants were asked to rate how often they had experienced the symptomatology in the past 7 days, on a four-point Likert scale ranging from 0 = *rarely or none of the time (less than 1 day)* to 3 = *almost or all of the time (5–7 days)*. The total score ranges from 0 to 60, with a higher score reflecting greater severity. The CES-D is composed of four dimensions: somatic symptoms, depressed affect, (lack of) positive affect and interpersonal problems. A score of 16 or more is suggestive of the presence of depressive symptoms (probable depression), and a score of 25 or more is highly associated with major depression. In this study, Cronbach's *α* was 0.92 at baseline.

#### Secondary outcomes

*Anxiety.* The seven-item General Anxiety Disorder Scale (GAD) was used to measure anxiety (Spitzer *et al*., 2006). Each item was rated on a four-point Likert scale ranging from 0 = *never* to 3 = *often (almost every day)*. The Chinese version of the GAD has been used among PLWH in China (Qiu *et al*., 2014a, 2014b). The cut-off point of 5 or above indicates a probable case of anxiety disorder. In this study, Cronbach's *α* was 0.93 at baseline.

*Positive affect and negative affect* were assessed by the 20-item Positive and Negative Affect Schedule (PANAS; Watson *et al*., 1988). As pointed out by Watson and Clark (1997), positive affect and negative affect are two distinct constructs rather than two extremes of the same dimension (Watson and Clark, 1997). PANAS has been used in the Chinese population (with Cronbach's *α* of 0.85 for the Positive Affect subscale and 0.83 for the Negative Affect subscale; Huang *et al*., 2003). It consists of 20 items, of which ten items reflect expectations for positive affect and ten for negative affect. The participants were asked to rate to what extent they currently felt a particular way on a five-point Likert scale ranging from 1 = *very slightly or not at all* to 5 = *extremely*. Scores for each subscale range from 10 to 50, with higher scores representing higher levels of positive affect and negative affect. In this study, Cronbach's *α* was 0.87 for the Positive Affect subscale and 0.93 for the Negative Affect subscale at baseline.

*Gratitude* was assessed by the six-item Gratitude Questionnaire (Emmons *et al*., 2003). Each item was rated on a seven-point Likert scale ranging from 1 = *strongly disagree* to 7 = *strongly agree*. In this study, the Cronbach's *α* value for the six-item scale was 0.52. Deletion of the last item (‘Long amounts of time can go by before I feel grateful to something or someone’) improved reliability (the Cronbach's *α* value at baseline increased to 0.80). The resulting modified five-item version was therefore used in the data analysis.

*Happiness.* The four-item validated the Subjective Happiness Scale (SHS) was used to measure general happiness (Lyubomirsky and Lepper, 1999). Each item was rated on a seven-point Likert scale. In this study, Cronbach's *α* was 0.79 at baseline.

*Social support.* Two items were constructed for this study to gauge the level of emotional and material social support that the participants were receiving. The participants were asked the following questions: (1) ‘How much support can you obtain from family/friends/colleagues when you need to talk or to obtain emotional support?’ and (2) ‘How much support can you obtain from family/friends/colleagues when you need material support (e.g. financial help)?’ Responses were recorded on an 11-point scale ranging from 0 = *none* to 10 = *tremendous*. The Cronbach's *α* for the scale obtained by summing the responses to the two items was 0.71 at baseline.

#### Intervention adherence

The participants were asked at M1 about the frequency that they performed the TGT exercise (TGT-SN and TGT-only groups), reviewed others' messages (TGT-SN group; the options were <1, 1, 2, 3–4, 5–6 or 7 days/week), and provided others with positive feedback (TGT-SN group; the options were ⩽10, 11–20, 21–30, 31–40, 41–50, 51–60 or >60 positive feedback/week).

#### Process evaluation

Information on detecting potential contamination (e.g. ‘Have you participated in other psychological counselling or intervention during the intervention period?’), and on the perceived effectiveness of the intervention (e.g. ‘To what extent was the TGT exercise effective in promoting your mental health’) was collected.

### Sample size and power

The primary outcome of this study was probable cases of mild-to-severe depression in the HIVMSM. Based on a previous study, about 48% of the HIVMSM in China would qualify as probable cases of mild-to-severe depression (Wu, 2012). The target sample size of this study was 400 HIVMSM (133 HIVMSM per study arm). This sample size allowed for the detection of the smallest between-group difference (TGT-SN *v*. the control group on absolute risk reduction) of 20% in the prevalence of the primary outcome, allowing for a 30% drop-out rate. The power and significance level were set to 0.8 and 0.05, respectively. Our final sample of 404 participants exceeded this target size.

### Statistical analysis

We used *χ*^2^ tests for the analysis of categorical variables and a one-way ANOVA for continuous variables to examine between-group differences at various time points. To examine the overall effect of TGT-SN and TGT-only with reference to the control group, controlling for potential covariates, repeated-measures analyses were conducted by using generalised estimation equations (GEEs). First, we entered the main effect of the intervention (TGT-SN and TGT-only *v*. control) and time in the model (Model 1); we then entered the main effect of the intervention, time and baseline outcome score (Model 2). Model 3 added an interaction term between intervention condition and time to Model 2. All statistical tests were two-sided and a *p*-value of <0.05 was considered statistically significant.

## Results

### Baseline background characteristics of the participants

The baseline differences in background characteristics (i.e. socio-demographic characteristics, HIV-related characteristics and social-psychological outcomes) across the three groups were not statistically different (Table 1). Among all participants, the majority (98.0%) were of Han ethnicity; 61.6% were aged 30 years or under; 61.1% had attended university or above; 51.5% had had monthly personal income ≤RMB 3000 (about US$480); 89.4% were HIV asymptomatic; <10% had never taken a CD4 test; 35.4% and 62.1% had not disclosed their HIV status to any male regular partners and non-regular sex partners, respectively. The prevalence of mild-to-severe depression and anxiety was 55.9% and 50.7%, respectively.

**Table 1.** Participants' baseline characteristics by intervention groups

	All (*N* = 404)	Control (*N* = 136)	PPI only (*N* = 139)	PPI-SN (*N* = 129)	p*
	*n* (%)	*n* (%)	*n* (%)	*n* (%)	
*Demographic characteristics*					
Ethnic					
Han	396 (98.0%)	133 (97.8%)	138 (99.3%)	125 (96.9%)	0.366
Others	8 (2.0%)	3 (2.2%)	1 (0.7%)	4 (3.1%)	
Hukou					
Chengdu	224 (55.4%)	77 (56.6%)	74 (53.2%)	73 (56.6%)	0.811
Others	180 (44.6%)	59 (43.4%)	65 (46.8%)	56 (43.4%)	
Age					
⩽30	249 (61.6%)	81 (59.6%)	91 (65.5%)	77 (59.7%)	0.517
>30	155 (38.4%)	55 (40.4%)	48 (34.5%)	52 (40.3%)	
Education level					
Below university	157 (38.9%)	61 (44.9%)	49 (35.3%)	47 (36.4%)	0.208
University or above	247 (61.1%)	75 (55.1%)	90 (64.7%)	82 (63.6%)	
Marital status					
Single	262 (64.9%)	93 (68.4%)	89 (64.0%)	80 (62.0%)	0.122
Married/cohabiting with girlfriend	37 (9.2%)	16 (11.8%)	7 (5.0%)	14 (10.9%)	
Cohabiting with boyfriend	79 (19.6%)	20 (14.7%)	35 (25.2%)	24 (18.6%)	
Divorced/widow/others	25 (6.2%)	7 (5.1%)	7 (5.0%)	11 (8.5%)	
Job					
Full-time	275 (68.1%)	96 (70.6%)	87 (62.6%)	92 (71.3%)	0.230
Part-time/unemployed	129 (31.9%)	40 (29.4%)	52 (37.4%)	37 (28.7%)	
Income					
⩽3000	208 (51.5%)	70 (51.5%)	69 (49.6%)	69 (53.5%)	0.820
>3000	196 (48.5%)	66 (48.5%)	70 (50.4%)	60 (46.5%)	
Sexual orientation					
Homosexual	344 (85.1%)	110 (80.9%)	123 (88.5%)	111 (86.0%)	0.195
Heterosexual/bisexual/not sure	60 (14.9%)	26 (19.1%)	16 (11.5%)	18 (14.0%)	
*HIV-related characteristics*					
HIV stage					
Asymptomatic HIV infection	361 (89.4%)	124 (91.2%)	123 (88.5%)	114 (88.4%)	0.699
AIDS	43 (10.6%)	12 (8.8%)	16 (11.5%)	15 (11.6%)	
Ever conducted CD4 test					
Yes	372 (92.1%)	125 (91.9%)	124 (89.2%)	123 (95.3%)	0.177
No	32 (7.9%)	11 (8.1%)	15 (10.8%)	6 (4.7%)	
Disclosure to RP					
Have disclosed to all	172 (42.6%)	56 (41.2%)	62 (44.6%)	54 (41.9%)	0.936
Have disclosed to some	89 (22.0%)	33 (24.3%)	28 (20.1%)	28 (21.7%)	
Have disclosed to none	143 (35.4%)	47 (34.6%)	49 (35.3%)	47 (36.4%)	
Disclosure to NRP					
Have disclosed to all	82 (20.3%)	21 (15.4%)	30 (21.6%)	31 (24.0%)	0.432
Have disclosed to some	71 (17.6%)	27 (19.9%)	25 (18.0%)	19 (14.7%)	
Have disclosed to none	251 (62.1%)	88 (64.7%)	84 (60.4%)	79 (61.2%)	
Disclosure to female sexual partners					
Have no female sexual partners	304 (75.2%)	96 (70.6%)	114 (82.0%)	94 (72.9%)	0.345
Have disclosed to all	24 (5.9%)	11 (8.1%)	4 (2.9%)	9 (7.0%)	
Have disclosed to some	15 (3.7%)	5 (3.7%)	4 (2.9%)	6 (4.7%)	
Have disclosed to none	61 (15.1%)	24 (17.6%)	17 (12.2%)	20 (15.5%)	
Self-perceived health					
Very poor/poor	31 (7.7%)	14 (10.3%)	10 (7.2%)	7 (5.4%)	0.319
Fair/good/very good	373 (92.3%)	122 (89.7%)	129 (92.8%)	122 (94.6%)	
*Mental health*					
Probable cases of depression (CESD)					
No	178 (44.1%)	53 (39.0%)	68 (48.9%)	57 (44.2%)	0.251
Yes	226 (55.9%)	83 (61.0%)	71 (51.1%)	72 (55.8%)	
Probable cases of anxiety (GAD)					
No	199 (49.3%)	63 (46.3%)	71 (51.1%)	65 (50.4%)	0.698
Yes	205 (50.7%)	73 (53.7%)	68 (48.9%)	64 (49.6%)	
	Mean (s.d.)	Mean (s.d.)	Mean (s.d.)	Mean (s.d.)	
*Social-psychological factors*					
Negative affect	22.94 (8.70)	23.80 (9.03)	22.45 (8.31)	22.57 (8.76)	0.365
Happiness	16.91 (5.05)	16.40 (5.05)	17.55 (5.21)	16.77 (4.84)	0.152
Gratitude	23.76 (5.71)	22.89 (6.22)	24.30 (5.24)	24.09 (5.59)	0.089
Positive affect	27.19 (7.32)	26.81 (6.91)	28.16 (7.20)	26.56 (7.80)	0.152
Social support	12.60 (4.99)	12.29 (5.15)	12.63 (4.69)	12.91 (5.16)	0.592

*p-value by *χ*^2^ test for categorical variable and Kruskal–Wallis one-way ANOVA for continuous variables.

### The primary outcome

The prevalence of probable depression (CESD ⩾ 16) was 55.8%, 51.1% and 61.0% in the TGT-SN, TGT-only and control groups at baseline (M0), respectively (*χ*^2^ test, *p* = 0.251; Table 1). The prevalence of probable depression among the TGT-SN and TGT-only groups was lower than that of the control group but the differences were not statistically significant (M1: 48.4% and 47.5% *v*. 54.8%; M4: 51.9% and 50.4% *v*. 61.9%; M7: 48.4% and 47.8% *v*. 58.3%; M13: 45.5% and 54.2% *v*. 55.6%; *p*s = 0.117–0.423, *χ*^2^ test; Table 2).

**Table 2.** Comparison of between-group and within-group differences in the prevalence of probable cases of depression and anxiety for TGT-SN, TGT-only and control group over the study period

	Control		TGT-SN		TGT-only		Overall between-group	TGT-SN *v*. control	TGT-only *v*. control	TGT-SN *v*. TGT-only
	%	*p* value (McNemar test)	%	*p* value (McNemar test)	%	*p* value (McNemar test)	*p* value (*χ*^2^ test)	*p* value (*χ*^2^ test)	*p* value (*χ*^2^ test)	*p* value (*χ*^2^ test)
Depression										
Baseline (M0)	61.0%		55.8%		51.1%					
M1	54.8%	0.291	48.4%	0.211	47.5%	0.615	0.423	0.301	0.225	0.876
M4	61.9%	0.885	51.9%	0.560	50.4%	1.000	0.117	0.101	0.054	0.796
M7	58.3%	0.766	48.4%	0.262	47.8%	0.597	0.158	0.110	0.084	0.924
M13	55.6%	0.883	45.5%	0.108	54.2%	0.771	0.261	0.130	0.830	0.189
Anxiety										
Baseline (M0)	53.7%		49.6%		48.9%					
M1	54.8%	0.890	43.0%	0.253	43.9%	0.443	0.097	0.055	0.070	0.880
M4	60.4%	0.298	42.6%	0.289	47.5%	0.901	**0**.**011**	**0**.**004**	**0**.**032**	0.426
M7	59.8%	0.410	46.8%	0.784	46.4%	0.807	**0**.**045**	**0**.**036**	**0**.**027**	0.942
M13	51.3%	1.000	43.8%	0.471	54.2%	0.461	0.265	0.254	0.657	0.113

Bold significant p < 0.05; underline marginal significant results with p value less than 0.1 but greater than 0.05.

The GEE analysis showed that the main effects of TGT-SN (OR = 0.75, 95% CI 0.52–1.09, *p* = 0.131; Table 4) and TGT-only (OR = 0.83, 95% CI 0.57–1.21, *p* = 0.332; Table 4) in reducing depression were not statistically significant when controlling for baseline depression score. The interactions between the intervention groups (TGT-SN and TGT-only) and time also indicated no significant differences in treatment effects over time (*p*s = 0.511–0.542).

### Secondary outcomes

The comparison of between-group and within-group differences in continuous secondary outcome variables for TGT-SN, TGT-only and control group over the study period was shown in Table 3. The GEE analysis (Model 2) showed a significant main effect of TGT-SN *v*. control in reducing the odds of anxiety (adjusted OR = 0.62, 95% CI 0.43–0.89, *p* = 0.009; Table 4), and level of negative affect (*β* = −1.62, 95% CI −2.98 to −0.26, *p* = 0.019; Table 4), when controlling for baseline anxiety or negative affect scores, respectively. The participants in the TGT-SN group thus showed significantly lower anxiety symptoms and less negative affect over time compared with those in the control group. There were no significant effects for TGT-SN in the other secondary outcomes (i.e. gratitude, positive affect, subjective happiness and social support). For the TGT-only group, no significant main effect was found for any secondary outcomes in the adjusted GEE models.

**Table 3.** Comparison of between-group and within-group differences in secondary outcome variables (continuous) for TGT-SN, TGT-only and control group over the study period

Measures	Control		TGT-SN		TGT-Only		Overall between-group difference^a^		TGT-SN, *v*. control	TGT-only *v*. control	TGT-SN *v*. TGT-only
	Mean (s.d.)	Change from M0, mean (s.d.)^b^	Mean (s.d.)	Change from M0, mean (s.d.)^b^	Mean (SD)	Change from M0, mean (s.d.)^b^	*F*	*p*	*p* value	*p* value	*p* value
Gratitude											
M0	22.89 (6.22)		24.09 (5.59)		24.30 (5.24)						
M1	20.86 (4.82)	2.07 (6.30)***	21.86 (5.68)	2.26 (6.19)***	22.21 (5.32)	2.09 (6.45)***	2.392	0.093	0.126	**0**.**035**	0.590
M4	21.30 (4.42)	1.65 (6.11)**	21.71 (4.13)	2.39 (6.36)***	22.60 (4.54)	1.70 (5.94)***	3.193	**0**.**042**	0.451	**0**.**014**	0.094
M7	22.20 (5.03)	0.71 (6.16)	22.71 (4.21)	1.40 (6.28)*	22.91 (5.12)	1.39 (6.24)**	0.752	0.472	0.404	0.233	0.737
M13	22.68 (4.54)	0.89 (5.98)	23.87 (4.70)	0.35 (5.88)	23.60 (4.39)	0.83 (5.91)	2.189	0.114	**0**.**048**	0.118	0.656
PA											
M0	26.81 (6.91)		26.56 (7.80)		28.16 (7.20)						
M1	25.43 (5.73)	1.39 (7.94)*	26.06 (7.06)	0.41 (8.44)	26.86 (6.34)	1.29 (7.68)*	1.736	0.178	0.422	0.064	0.307
M4	24.05 (6.59)	2.86 (7.63)***	23.90 (5.86)	2.66 (8.70)***	24.88 (5.29)	3.27 (7.35)***	1.088	0.338	0.834	0.247	0.175
M7	25.86 (6.95)	0.99 (8.35)	25.62 (6.58)	0.97 (8.27)	26.42 (6.06)	1.74 (7.92)*	0.526	0.591	0.764	0.485	0.320
M13	26.45 (6.64)	0.59 (7.97)	26.51 (6.72)	−0.45 (8.63)	27.81 (6.09)	0.51 (7.93)	1.658	0.192	0.951	0.108	0.127
SHS											
M0	16.40 (5.05)		16.77 (4.84)		17.55 (5.21)						
M1	16.19 (4.01)	0.24 (4.91)	16.82 (4.34)	−0.08 (4.94)	17.70 (4.12)	−0.14 (5.09)	4.586	**0**.**011**	0.216	**0**.**003**	0.085
M4	16.40 (3.55)	0.04 (4.64)	16.63 (3.38)	0.14 (4.88)	17.22 (3.49)	0.33 (4.94)	2.065	0.128	0.588	**0**.**050**	0.162
M7	17.02 (4.07)	−0.65 (4.95)	17.07 (3.59)	−0.33 (5.09)	17.56 (4.25)	−0.01 (5.48)	0.743	0.477	0.922	0.271	0.323
M13	17.25 (4.14)	−0.41 (4.94)	17.62 (3.78)	−0.97 (4.70)*	17.56 (4.20)	0.02 (4.93)	0.277	0.758	0.492	0.555	0.914
Social support											
M0	12.29 (5.15)		12.91 (5.16)		12.63 (4.69)						
M1	13.21 (3.74)	−0.90 (5.18)*	13.59 (4.22)	−0.66 (5.35)	13.34 (3.82)	−0.71 (4.93)	0.328	0.720	0.425	0.783	0.595
M4	12.32 (4.16)	−0.02 (5.02)	12.77 (4.14)	0.15 (5.98)	12.98 (3.37)	−0.35 (5.03)	1.008	0.366	0.353	0.164	0.658
M7	12.23 (4.23)	0.02 (4.95)	12.56 (3.75)	0.32 (5.45)	12.99 (3.13)	−0.34 (4.85)	1.382	0.252	0.490	0.099	0.349
M13	12.11 (3.82)	0.29 (4.73)	13.03 (4.00)	−0.30 (5.56)	12.63 (3.16)	0.04 (4.71)	1.795	0.168	0.060	0.282	0.405
NA											
M0	23.80 (9.03)		22.57 (8.76)		22.45 (8.31)						
M1	19.23 (6.38)	4.56 (10.27)***	18.38 (7.33)	4.24 (10.57)***	18.92 (6.82)	3.53 (10.03)***	0.513	0.599	0.317	0.709	0.522
M4	20.82 (7.26)	3.02 (10.29)***	18.65 (7.06)	3.91 (10.69)***	19.66 (8.49)	2.78 (11.08)**	2.653	0.072	**0**.**022**	0.211	0.280
M7	20.89 (7.96)	3.08 (10.65)**	18.91 (7.84)	3.85 (11.18)***	18.82 (7.77)	3.72 (9.94)***	2.921	0.055	**0**.**044**	**0**.**031**	0.923
M13	21.36 (8.73)	1.87 (11.40)	19.09 (8.03)	3.36 (9.77)***	21.19 (9.01)	1.48 (10.84)	2.460	0.087	**0**.**047**	0.881	0.064

CESD, Center for Epidemiologic Studies Depression Scale; GAD, General Anxiety Disorder Scale; PA, Positive Affect Subscale; NA, Negative Affect Subscale; SHS, Subjective Happiness Scale; SWLS, Satisfaction with Life Scale.

a One-way ANOVA test.

b Paired *t*-test.

*p < 0.05; **p < 0.01; ***p < 0.001.

**Table 4.** Generalised estimation equations analyses predicting depression and anxiety and other secondary outcomes (continuous) at M1, M4, M7 and M13

Variable	Model 1 (unadjusted)		Model 2 (adjusted)		Model 3 (adjusted)	
	OR (95% CI)	*p*	AOR (95% CI)	*p*	AOR (95% CI)	*p*
*Primary outcome (categorical)*						
Depression						
TGT-SN *v*. control	0.69 (0.48, 1.01)	0.057	0.75 (0.52, 1.09)	0.131	0.87 (0.49, 1.54)	0.636
TGT-only *v*. control	0.73 (0.50, 1.05)	0.092	0.83 (0.57, 1.21)	0.332	0.71 (0.41, 1.24)	0.232
Time	1.01 (0.94, 1.08)	0.841	1.01 (0.94, 1.09)	0.749	1.01 (0.88, 1.16)	0.877
Baseline depression	N.A.	N.A.	1.04 (1.03, 1.06)	<0.001	1.04 (1.03, 1.06)	<0.001
TGT-SN × Time	N.A.	N.A.	N.A.	N.A.	0.94 (0.77, 1.14)	0.542
TGT-only × Time	N.A.	N.A.	N.A.	N.A.	1.06 (0.88, 1.28)	0.511
*Secondary outcomes*						
Anxiety (categorical)						
TGT-SN *v*. control	**0.60** (**0.42, 0.86)**	**0.006**	**0.62** (**0.43, 0.89)**	**0**.**009**	0.53 (0.29, 0.97)	0.038
TGT-only *v*. control	**0.70** (**0.49, 0.99)**	**0.045**	0.73 (0.51, 1.04)	0.083	0.51 (0.29, 0.89)	0.018
Time	1.04 (0.96, 1.13)	0.386	1.04 (0.96, 1.13)	0.335	0.97 (0.84, 1.12)	0.690
Baseline GAD score	N.A.	N.A.	1.07 (1.04, 1.10)	<0.001	1.07 (1.04, 1.10)	<0.001
TGT-SN × Time	N.A.	N.A.	N.A.	N.A.	1.06 (0.86, 1.32)	0.579
TGT-only × Time					1.16 (0.95, 1.42)	0.144
*Other secondary outcomes (continuous)*	*B* (95% CI)	*p*	*B* (95% CI)	*p*	*B* (95% CI)	*p*
Negative affect (NA)						
TGT-SN *v*. control	**−1.80** (**−3.18, −0.41)**	**0.011**	**−1.62** (**−2.98, −0.26)**	**0**.**019**	−0.56 (−2.53, 1.41)	0.576
TGT-only *v*. control	−0.95 (−2.40, 0.50)	0.199	−0.76 (−2.18, 0.66)	0.296	−0.46 (−2.36, 1.43)	0.631
Time	0.49 (0.19, 0.79)	0.001	0.50 (0.20, 0.80)	0.001	0.68 (0.13, 1.23)	0.016
Baseline NA score	N.A.	N.A.	0.16 (0.08, 0.23)	<0.001	0.16 (0.08, 0.23)	<0.001
TGT-SN × Time	N.A.	N.A.	N.A.	N.A.	−0.43 (−1.20, 0.33)	0.265
TGT-only × Time	N.A.	N.A.	N.A.	N.A.	−0.12 (−0.85, 0.61)	0.747
Gratitude						
TGT-SN *v*. control	0.76 (−0.16, 1.69)	0.107	0.48 (−0.37, 1.33)	0.267	0.25 (−1.05, 1.55)	0.709
TGT-only *v*. control	**1.08** (**0.15, 2.01)**	**0.023**	0.74 (−0.10, 1.58)	0.083	1.12 (−0.12, 2.36)	0.077
Time	0.59 (0.43, 0.75)	<0.001	0.57 (0.41, 0.72)	<0.001	0.59 (0.32, 0.86)	<0.001
Baseline gratitude score	N.A.	N.A.	0.27 (0.20, 0.34)	<0.001	0.27 (0.20, 0.34)	<0.001
TGT-SN × Time	N.A.	N.A.	N.A.	N.A.	0.09 (−0.30, 0.49)	0.639
TGT-only × Time	N.A.	N.A.	N.A.	N.A.	−0.16 (−0.53, 0.22)	0.420
Positive affect (PA)						
TGT-SN *v*. control	0.07 (−1.14, 1.28)	0.910	0.20 (−0.92, 1.31)	0.729	0.55 (−1.18, 2.28)	0.535
TGT-only *v*. control	1.03 (−0.07, 2.14)	0.067	0.69 (−0.33, 1.70)	0.183	0.84 (−0.78, 2.47)	0.310
Time	0.39 (0.16, 0.63)	0.001	0.39 (0.16, 0.63)	0.001	0.46 (0.05, 0.87)	0.027
Baseline PA score	N.A.	N.A.	0.27 (0.19, 0.34)	<0.001	0.27 (0.19, 0.34)	<0.001
TGT-SN × Time	N.A.	N.A.	N.A.	N.A.	−0.14 (−0.71, 0.43)	0.622
TGT-only × Time	N.A.	N.A.	N.A.	N.A.	−0.06 (−0.64, 0.51)	0.828
Subjective happiness (SHS)						
TGT-SN *v*. control	0.32 (−0.42, 1.06)	0.393	0.25 (−0.38, 0.88)	0.435	0.40 (−0.58, 1.38)	0.427
TGT-only *v*. control	**0.81** (**0.05, 1.58)**	**0.038**	0.48 (−0.17, 1.13)	0.150	1.36 (0.43, 2.30)	0.004
Time	0.21 (0.08, 0.35)	0.002	0.20 (0.07, 0.34)	0.003	0.35 (0.13, 0.57)	0.002
Baseline SHS score	N.A.	N.A.	0.32 (0.25, 0.38)	<0.001	0.32 (0.25, 0.38)	<0.001
TGT-SN × Time	N.A.	N.A.	N.A.	N.A.	−0.06 (−0.39, 0.27)	0.717
TGT-only × Time	N.A.	N.A.	N.A.	N.A.	**−0.36** (**−0.69, −0.03)**	**0**.**031**
Social support						
TGT-SN *v*. control	0.50 (−0.26, 1.26)	0.194	0.36 (−0.31, 1.03)	0.297	−0.04 (−1.04, 0.95)	0.932
TGT-only *v*. control	0.51 (−0.19, 1.21)	0.150	0.43 (−0.18, 1.03)	0.171	0.10 (−0.84, 1.03)	0.842
Time	−0.25 (−0.38, −0.12)	<0.001	−0.25 (−0.38, −0.12)	<0.001	−0.35 (−0.58, −0.12)	0.002
Baseline social support score	N.A.	N.A.	0.26 (0.19, 0.33)	<0.001	0.26 (0.19, 0.33)	<0.001
TGT-SN × Time	N.A.	N.A.	N.A.	N.A.	0.16 (−0.17, 0.50)	0.335
TGT-only × Time	N.A.	N.A.	N.A.	N.A.	0.13 (−0.16, 0.43)	0.375

### Intervention adherence and process evaluation

The compliance with the interventions was generally satisfactory. Of the 129 participants in the TGT-SN group, 89 (69.0%) conducted the TGT exercise at least 1 day per week, and 20 (15.5%) did so more than 5 days per week. Concerning feedback messages posted by others, 89.1% reviewed such messages, and 68.2% posted positive feedback to such messages. Of the 139 participants in the TGT-only group, 82 (59.0%) conducted the TGT exercise at least 1 day per week; 16 (11.5%) more than 5 days per week.

Among all participants, only nine (2.2%) participated in other psychological counselling or interventions during the intervention period (representing potential external contamination). Among the participants in the TGT-SN group, 43 (33.6%) communicated with other group members on matters beyond PPI via QQ.

Among the participants in the TGT-SN and TGT-only groups, respectively, 21.6% and 20.9% thought that the TGT exercise was ineffective in improving mental health, and 66.4% and53.4% expressed that the TGT exercise was easy to conduct.

### Serious adverse events

No observable adverse event was reported during the intervention period.

## Discussion

To our knowledge, this study is the first randomised controlled trial to investigate the efficacy of TGT in improving mental health among the PLWH population. We found that participation in TGT-SN was effective in reducing anxiety and negative affect among HIVMSM compared with the control group. The TGT-only group showed only numerically, but not statistically significant, less depression, anxiety and negative affect, compared with the control group. Neither intervention group (TGT-SN nor TGT-only) showed a statistically significant enhancement of positive well-being (i.e. gratitude, positive affect and happiness) among HIVMSM.

In the GEE analysis that controlled for baseline outcome values, the participants in TGT-SN showed less anxiety and negative affect. This corroborates the results of previous studies investigating the effects of TGT conducted among Internet-based samples (Seligman *et al*., 2005; Gander *et al*., 2013). This finding is also consistent with the results of a meta-analysis (Bolier *et al*., 2013) of the effects of PPIs among both clinical and non-clinical populations. Furthermore, it is interesting to note that the TGT-only intervention did not significantly improve mental health compared with the control.

The present study did not find any effect of TGT-SN or TGT-only in enhancing positive well-being, i.e. gratitude, positive affect and happiness, among HIVMSM, when adjusted for baseline score. This finding contrasts with previous studies that have found significant effects of TGT in enhancing happiness and positive affect among the general population (Emmons and McCullough, 2003; Seligman *et al*., 2005; Mongrain and Anselmo-Matthews, 2012; Gander *et al*., 2013). There are several possible explanations. First, the effect of TGT in enhancing positive well-being may be population-specific and may not apply to the Chinese population. The literature gives an important role to religion in shaping feelings of gratitude in life. People who regularly attend religious services or engage in religious practices are more likely to have a higher level of gratitude in all aspects of life (McCullough *et al*., 2002; Emmons and Keneezel, 2005; Krause, 2009). The majority (89.6%) of people in China are not religious (Lu, 2014); thus it might be more difficult to establish and enhance gratitude among the Chinese population than among people in countries with higher rates of religiosity. Second, cultivating positive feelings in Chinese culture may be more difficult as the tradition teaches people to be humble and not to disclose their positive feelings. Third, the norms for positive emotions are different across various nations (Eid and Diener, 2001). In China, some individuals believe that positive emotions are undesirable. The Chinese have reported lower frequency and intensity scores for positive affect than those reported in the USA and Australia (Eid and Diener, 2001). Fourth, it is possible that the dosage was not high enough to increase positive well-being. Although the majority of the participants in TGT-SN (about 70%) and TGT-only (about 60%) recorded three good things messages on at least 1 day per week, compliance with the daily exercise we requested was low. Less than 20% of the participants in TGT-SN and TGT-only conducted the exercise daily. It is possible that daily exercise for a month was too burdensome for the participants.

In this study, we expected to find that social networks could act as a platform/system for the participants to seek social/peer support. However, no improvement in social support was observed in the TGT-SN group over the study period. This may be partially due to the participants having been requested to post only supportive messages and not to air negative views or personal problems in the posted messages. However, in addition to positive reinforcement, sharing personal experiences/problems and providing advice are important empowering processes through which online support groups may improve mental health among PLWH (Mo and Coulson, 2012, 2013, 2014). Indeed, people will only perceive that they are supported when they encounter problems. The restriction on disclosing negative views or weaknesses might have prevented the participants from perceiving a higher level of support from their social network. Another possible explanation is that the measurements of social support used in this study were not suited to capture the positive reinforcement obtained from group members. The social support scale used in this study assessed the participants' levels of emotional and instrumental social support obtained from their family/friends/colleagues, rather than the positive reinforcement obtained from their group members. Thus, the measure may not have been accurate in establishing the changes in perceived social support obtained through positive reinforcement.

A high prevalence of mental health problems was found among the HIVMSM in this study. Among all participants at baseline, 55.9% had mild-to-severe depression, and 50.7% had anxiety. These results are consistent with those of previous studies conducted among PLWH in China (Molassiotis *et al*., 2002; Su *et al*., 2013) and suggest a significant threat to HIVMSM, given that since such mental health problems are associated with a number of HIV-related health outcomes and high-risk behaviour, such as low service utilisation rate, poor ART (Gordillo *et al*., 1999; Catz *et al*., 2000; Langebeek *et al*., 2014), faster progression to AIDS and shorter survival (Perry and Fishman, 1993; Lyketsos *et al*., 1996; Cook *et al*., 2002; Schuster *et al*., 2012), substance use and the practice of unsafe sex (Parsons *et al*., 2003). Mental health services should be provided to reduce mental health problems among PLWH including HIVMSM. In developed countries, such as the USA and the UK, mental health screening and support services have been integrated into the HIV/AIDS care system (AIDS Institute New York State Department of Health, 2012; British Psychological Society *et al*., 2011). However, there is a shortage of mental health promotion and treatment services among PLWH, including HIVMSM, in most parts of China (Zhao *et al*., 2009; Tao *et al*., 2010). One study found that <9% of PLWH in China who were depressed had ever received treatment for the condition (Jin *et al*., 2006). The findings of our study suggest that developing integrated mental health prevention, screening and treatment services as an integral part of the HIV/AIDS care system is warranted in China.

There are several limitations to this study. First, selection bias may be present and there were non-contacts. Second, reporting bias may be present due to the nature of self-reported data. Third, this study was conducted only among HIVMSM in one city. The results cannot be generalised to HIVMSM in different parts of China or other PLWH populations. Fourth, the measurement of social support was created for this study and had not been previously validated. Fifth, the sample size may not be large enough to detect significant differences in some outcomes; the power for depression outcomes is limited. Future studies with a larger sample size may demonstrate more powerful effects. Finally, the rate of compliance with our instructions was quite low. However, it is impossible to compare the compliance rate in this study with previous studies, because compliance with TGT exercises was not well documented in previously published studies and the settings were different (Emmons and McCullough, 2003; Seligman *et al*., 2005; Mongrain and Anselmo-Matthews, 2012; Gander *et al*., 2013).

## Conclusion

In summary, this study is the first randomised controlled trial investigating the effect of a PPI on mental health among PLWH in China. A novel intervention combining the TGT exercise with electronic social networking, TGT-SN, was found effective in reducing anxiety and negative affect among HIVMSM. TGT-SN can easily be translated into regular services, as it can be delivered at no cost and does not require trained psychology professionals for administration and maintenance. It can also be easily scalable with the wide use of electronic social networking among HIVMSM in China. It is a sustainable intervention to fill the mental health service gap, especially in resource-limited settings.

## Data

The data-sets used and analysed in the current study are available from the corresponding author on reasonable request.
